# Immune checkpoint inhibitor-related myositis and myocarditis: diagnostic pitfalls and imaging contribution in a real-world, institutional case series

**DOI:** 10.1007/s00415-023-12134-x

**Published:** 2023-12-23

**Authors:** Alex Vicino, Andreas F. Hottinger, Sofiya Latifyan, Sarah Boughdad, Fabio Becce, John O. Prior, Thierry Kuntzer, Jean-Philippe Brouland, Vincent Dunet, Michel Obeid, Marie Théaudin

**Affiliations:** 1 Nerve-Muscle Unit, Neurology Service, Department of Clinical Neurosciences, Lausanne University Hospital and University of Lausanne, Lausanne, Switzerland; 2 Lundin Family Brain Tumor Center, Departments of Oncology and Clinical Neurosciences, Lausanne University Hospital and University of Lausanne, Lausanne, Switzerland; 3 Department of Oncology, Lausanne University Hospital and University of Lausanne, Lausanne, Switzerland; 4 Department of Nuclear Medicine and Molecular Imaging, Lausanne University Hospital and University of Lausanne, Lausanne, Switzerland; 5 Department of Diagnostic and Interventional Radiology, Lausanne University Hospital and University of Lausanne, Lausanne, Switzerland; 6 Pathology Institute, Lausanne University Hospital and University of Lausanne, Lausanne, Switzerland; 7 Immunology and Allergy Service, Lausanne University Hospital and University of Lausanne, Lausanne, Switzerland

**Keywords:** Immune-checkpoint-inhibitor, Immune-related-adverse-event, Myositis, Myocarditis, Imaging

## Abstract

**Background:**

Immune checkpoint inhibitors (ICIs) are reshaping the prognosis of many cancers, but often cause immune-related adverse events (irAEs). Among neurological irAEs, myositis is the most frequently reported. Our aim is to describe clinical and non-clinical characteristics, treatment and outcome of all irMyositis (skeletal limb-girdle and/or ocular myositis) and irMyocarditis cases in our reference center.

**Methods:**

We retrospectively enrolled all irMyositis/irMyocarditis patients seen between 2018 and 2022. We reviewed demographics, clinical characteristics, biological, neurophysiological, imaging workup, treatment and outcome.

**Results:**

We included 14 consecutive patients. The most frequent treatments were pembrolizumab (35%) or ipilimumab–nivolumab combination (35%). Limb-girdle, ocular (non-fluctuating palpebral ptosis and/or diplopia with or without ophthalmoparesis) and cardiac phenotypes were equally distributed, overlapping in 40% of cases. Ocular involvement was frequently misdiagnosed; review of brain MRIs disclosed initially missed signs of skeletal myositis in one patient and ocular myositis in 3. Seven patients had other co-existing irAEs. When performed, myography showed a myogenic pattern. CK was elevated in 8/15 patients, troponin-T in 12/12 and troponin-I in 7/9 tested patients. ICI were discontinued in all cases, with further immunosuppressive treatment in nine patients. In most cases, neurological and cardiological outcome was good at last follow-up.

**Conclusion:**

Myositis is a potentially severe irAE. Despite its heterogeneous presentation, some highly suggestive clinical symptoms, such as ocular involvement, or radiological signs should raise physicians’ attention to avoid misdiagnosis. We thus recommend a multidisciplinary assessment (including complete neuromuscular evaluation) even in case of isolated myocarditis. Our series underlines the importance of an early diagnosis, since suspension of ICI and adequate treatment are usually associated with good functional outcome.

**Supplementary Information:**

The online version contains supplementary material available at 10.1007/s00415-023-12134-x.

## Introduction

Immune checkpoints (IC), including the cytotoxic T lymphocyte antigen 4 (CTLA-4) and programmed cell death protein 1 (PD-1)/programmed cell death ligand 1 (PD-L1) signaling pathways, are key regulators of immune homeostasis through the downregulation of T-cell responses and immune activation [1, 2]. Currently approved ICIs target the molecules CTLA4, PD-1, LAG3 and PD-L1. Indication of immunotherapy, initially limited to metastatic melanoma [3], has progressively extended to other cancers and ICI are now the new gold-standard therapy for several cancers [4]. In Switzerland, immune checkpoint inhibitors (ICI) were first approved in 2016 and their clinical use has become routine since 2017.

As a consequence of the inhibitory pathways to self-cell recognition blockade, a significant proportion of patients treated with ICIs experiences a spectrum of immune-related adverse events (irAEs) that may affect every organ [5, 6] and cause significant morbidity, limiting the use of these drugs and increasing costs to the healthcare system [7, 8].

Neurological irAEs account for 1% of all irAEs [8, 9]. Although rare, they can be responsible for severe complications, from disability to death. Myositis is a potentially disabling and life-threatening irAE, reported as the most common neurological irAE and accounting for 32% of all reported cases in the literature [10]. The most common clinical patterns are limb-girdle (51% of reported cases), ocular (43%), bulbar (46%), facial-neck (29%). The association of skeletal myositis with myocarditis is frequent (24%) and plays an important role in the management and prognosis of these patients. Overlap with irAE myasthenic syndromes is also reported [11].

When adequately treated, the outcome is usually favorable, but severe outcomes, including death, have been reported, mainly related to cardiac and/or respiratory involvement; myositis represents the third most common cause of death among neurological irAEs [10].

To better characterize the spectrum of irMyositits, we retrospectively reviewed the characteristics, management and outcome of patients with such diagnosis in our reference center.

## Patients and methods

### Population

We retrospectively included all patients diagnosed with irMyositis, (defined as skeletal axial and/or limb-girdle myositis, with or without ocular myositis) and irMyocarditis, at Lausanne University Hospital between 2018 and 2022. We identified patients from the databases of our nerve-muscle and neuro-oncology units and from the immunology and cardiology departments. Depending on the main clinical manifestations and hospital logistics at time of admission, patients care was under the responsibility of either the neurology (including both neuro-muscular and neuro-oncology units), cardiology or immunology departments. All patients were admitted for the work-up and management of their irAEs. Data were collected from the in-patient ward and ambulatory clinical charts.

### Clinical data

We reviewed patient demographics, cancer types, types of ICI used (monotherapy or combination), time from ICI administration and irAE onset. Based on reported symptoms and neurological clinical examination by attending physicians, we classified the clinical pattern of weakness as follows: limb-girdle, ocular, cardiac or a combination of these presentations. Skeletal myositis diagnosis was considered in the presence of objective and typical motor impairment, e.g. axial and/or symmetrical, proximal limb weakness (referred as “limb-girdle”) and elevation of muscle enzymes, by analogy with published idiopathic inflammatory myopathy criteria [12]. As discussed later, non-fluctuating ocular signs (palpebral ptosis, diplopia with or without visible ophthalmoparesis) are pathognomonic of irMyositis and were considered as strong supportive criteria for the diagnosis. When available, EMG abnormalities (recording of spontaneous activity and myogenic pattern on electromyography), radiological signs of muscle inflammation and histopathological evidence of myositis upon muscle biopsy were also considered as supportive diagnostic criteria. According to published criteria [13, 14], myocarditis was considered “definite” in the presence of clinical symptoms, elevation of cardiac enzymes and evidence of myocarditis on cardiac MRI and “possible” in the presence of clinical symptoms, elevated enzymes and electrocardiographic evidence of peri-myocarditis. Severity of symptoms at peak was quantified with the modified Rankin Scale (mRS) [13], scoring “0” if no residual symptom, “1” if minor symptoms without limitation for daily activities, “2” for minor disability (unable to perform some of the previous daily activities), “3” for moderate disability (patient able to walk without assistance), “4” in case of disability limiting personal activities or inability to walk unassisted, “5” for bedridden patients, and “6” for death (caused by the irAE).

For short as well as long-term outcome, we considered as minor symptoms (e.g. mRS 1) isolated ptosis, myalgia or fatigue without weakness when they had no impact on normal daily activities.

### Biological data

We collected creatinine kinase (CK), troponin T and troponin I available serum levels at peak. We also collected specific auto-immune laboratory results, including anti-acetylcholine receptor (anti-AchR), anti-muscle specific kinase antibodies (anti-MuSK) and “polymyositis” antibody panel, which includes anti-Jo1, anti-PL7, anti-PL12, anti-EJ, anti-SRP, anti-MI-2, anti-MDA5, anti-Tif1-gamma, anti-Ku, anti-PM-Scl, anti-Scl70 and anti-SSA 52 kD antibodies.

### Nerve conduction studies (NCS) and electromyography (EMG)

NCS and EMG were performed by senior neurologists in our neuromuscular unit. We noted the type of study performed (nerve conduction, 3 Hz repetitive stimulations, (EMG)) and their results (normal or not, if abnormal, type of abnormalities).

### Imaging and nuclear medicine data

We reviewed the results of all the imaging investigations performed in the diagnostic work-up or for the follow-up (of the irAE or the oncologic disease). Senior radiologists, radio-cardiologists or nuclear medicine physicians interpreted brain, muscle, and cardiac magnetic resonance imaging (MRI), and positron emission tomography (PET)/CT scan, respectively. The radiological work-up was tailored to the clinical and biological presentation. Brain and/or whole-body muscle MRI were performed in patients with ocular or neurological symptoms or as a follow-up for known brain tumor or metastasis. In case of myocardial inflammation suspicion, cardiac MRI or [^68^Ga] Ga-DOTATOC PET/CT-scan were performed. A senior neuroradiologist (VD) reviewed available brain MRI in patients without notion of ocular symptoms in order to look for signs of orbital inflammation possibly missed at first lecture, as well as meningeal or hypophyseal inflammatory signs. Diagnosis of orbital inflammation was made according to the following criteria: T2 hypersignal of oculomotor muscles (in comparison to the temporal muscle), T2 hypersignal of intraconal fat and enhancement after gadolinium injection.

### Pathology

We reviewed the results of available biopsies. Skeletal muscle biopsy samples were divided in three: the first part was frozen in isopentane and liquid azote and sliced (thickness 4 µm) in cryostat, then prepared with the following stains: hematoxylin–eosin (HE), Engel, ATPase at pH 4.2, NADH-TR, CD3, CD4, CD8, CD20, CD68, C5b-9, MHC1 (major histocompatibility complex I), TDP43, dysferlin. The second was fixed in formalin and included in a paraffin block and prepared with HE stain. The third one was directly frozen in liquid nitrogen for the tissue bank.

### Treatment and outcome

We noted if the immunotherapy was discontinued after irAE diagnosis and reviewed treatment used to manage the irAE. We quantified short-term functional outcome with the mRS. Long-term outcome was assessed at last available follow-up and took into account relapses of irAE after immunosuppression tapering, and survival. The functional scoring was based on the assessments by a neurologist, oncologist or physiotherapist, whether relapses and survival were collected from the last available medical chart.

### Statistical analysis

We reported patient characteristics as numbers and percentages for categorical variables and median (IQR, range) or mean (± SD) values, defining the minimum (min) and maximum (max) [min–max], for continuous variables as appropriate.

Difference between mean time from ICI initiation to cardiac or neurological symptom onset was estimated with Welch two-sample T-test.

### Ethics

The study protocol was approved by the regional ethic committee (*Commission Cantonale d'éthique de la recherche sur l'être humain - Vaud*), project ID 2023-00017. All patients signed a consent for data reuse.

### Data availability

Data are stored in Lausanne University Hospital’s database and are available upon reasonable request. All anonymized data from this study will be shared as aggregated data by request from any qualified investigator.

## Results (Table 1)

**Table 1:** Population characteristics, clinical presentation, biological values, radiological work-up, treatment and outcome

N°	Sex	Age	Cancer	ICI	∆ ICI (days)	Axial, limb-girdle	Ocular	Cardiac	Other	Extra-musc	mRS peak	CK	Trop T	Trop I	PM AB	B MRI	C MRI	M MRI	PET/CT	Trt	mRS post
1	M	74	Hepatocellular carcinoma Stage G2	ATE	75	Y	Ptosis, diplopia	N	Dysphagia	N	3	**331**	**1210**	30.7	NEG	OI	Normal	n/a	Myocardial uptake	IVIG, MP, PR	2
2	F	63	Glioblastoma OMS grade IV	NIV	41	Y	N	N	Myalgia	Cutaneous, hypothyroidism	2	24	18	n/a	NEG	Known tumor, no OI	n/a	Myositis, fasciitis	n/a	MP, PR	1
3	F	71	Serous ovarian FIGO IIIc	ATE	20	N	Diplopia	N	N	Colitis	1	26	n/a	n/a	NEG	OI	n/a	n/a	n/a	none	0
4	F	61	Melanoma Stage Ia	PEM	55	N	Diplopia	N	N	N	1	158	7	n/a	n/a	OI, and known metastasis	Normal	n/a	n/a	PR	0
5	M	64	Melanoma Stage IIIc	PEM	33	Y	N	Possible	Myalgia	Hepatitis	1	**7589**	**1231**	**88.7**	n/a	n/a	Normal	n/a	Myocardial uptake	MP, PR, MM, INFL	0
6	M	66	Prostatic adenocarcinoma Gleason 9	IPI-NIV	48	N	N	Possible	N	N	3	**524**	**2389**	**169.9**	n/a	n/a	n/a	n/a	Myocardial uptake	MP, MM, TOC	3
7	F	77	Merkel carcinoma Stage IIa	PEM	77	N	N	Possible	N	N	2	**142**	**229**	**n/a**	n/a	no OI	Pericarditis, no myocarditis	n/a	n/a	none	2
8	M	64	Epidermoid carcinoma In situ	PEM	64	Y	Diplopia	Possible	Myalgia	N	2	**4752**	**1556**	**1604.7**	n/a	n/a	Normal	n/a	Myocardial uptake	MP, PR	1
9	M	84	Melanoma Stage IV	NIV	84	Y	Diplopia	Probable	Myalgia	N	1	**386**	**933**	**212**	n/a	no OI; cervical myositis	Suggestive of myocarditis	n/a	Myocardial, axial uptake	PR	0
10	M	87	Desmoplastic melanoma Stage IV	IPI-NIV	87	N	N	Definite	N	Colitis	3	127	**996**	**5896.1**	n/a	n/a	Myocarditis	n/a	Myocardial uptake	MP, PR, TOC	1
11	M	56	Melanoma Stage IV	IPI-NIV	56	Y	Diplopia	N	N	Colitis	2	**235**	**40**	28	n/a	OI, and known metastasis	Normal	n/a	Myocardial uptake	MP, PR	1
12	M	64	Melanoma Stage IIIc	IPI-NIV	64	N	N	Possible	N	Cutaneous	1	101	**219**	**110**	n/a	No OI	n/a	n/a	Myocardial uptake	None	0
13	M	46	Clear cell renal carcinoma Fuhrmann grade II	PEM	46	Y	N	Definite	Myalgia	Thyroiditis, hepatitis	1	**2385**	**91**	**187**	n/a	n/a	Myocarditis	n/a	Myocardial uptake	None	0
14	F	43	Melanoma	IPI-NIV	52	N	Diplopia	N	Headache	Hypophisitis	1	**n/a**	**n/a**	**n/a**	n/a	OI, hypophisitis	n/a	n/a	n/a	None	0

Abnormal biological values are in bold

*ICI* immune checkpoint inhibitor; *∆ (delta)* time between ICI initiation and symptoms onset; *Cardiac* possible, probable or definite myocarditis diagnosis according to Bonaca criteria; *CK* creatinine kinase (N: 25–190 Units/liter); *Trop* Troponin T (N < 14 nanograms/liter); Troponin-I (N < 34.2 nanograms/liter). *PM AB* “polymyositis” antibody panel; *n/a* not available; Abnormal values in bold. *B MRI* brain magnetic resonance imaging; *OI* orbital inflammatory sign; *C MRI* cardiac MRI; *M MRI* muscle MRI; *PET-CT* [^68^Ga]-DOTATOC positron emission tomography-computed tomography; *FDG* fluorodeoxyglucose; *mRS* modified rankin scale (post: 30 days after symptom onset); *ATE*: atezolizumab; *PEM* pembrolizumab; *NIV* nivolumab; *IPI-NIV* ipilimumab-nivolumab combination; *IVIG* intravenous immunoglobulin; *MP* Methylprednisolone; *PR* prednisone; *INF* infliximab; *MM* mycophenolate mofetil; *TOC* tocilizumab; *extra musc* extra muscular

### Patients

Among the 16 patients who fulfilled the inclusion criteria, 14 were included (6 females, 35%). The remaining two patients declined consent for the reuse of their data and had passed away at time of the study. Median age at neurological presentation was 64 years (range 43–87 years). All patients had a malignant tumor with failure of first- line chemotherapies. The most frequent cancer was melanoma (6/14). All patients had metastatic cancer (except for one patient with glioblastoma, treated by nivolumab in the context of a clinical trial). The most frequent immunotherapies were ipilimumab and nivolumab combination (5/14) and pembrolizumab (5/14), followed by atezolimumab (2/14) and nivolumab (2/14). Patients developed the first muscle or cardiac symptom within a median time of 55 days (range 20–87 days) after initiation of ICI, with no significant difference between muscle and cardiac symptom onset. All patients were hospitalized at symptom onset, except for patient 2, who had recurrent episodes of myalgia, fever and weakness (first episode 41 days after ICI initiation) after each ICI cycle, and for whom the diagnosis was eventually made only 7 months after the first symptoms (233 days after ICI onset).

### Clinical characteristics of patients with irMyositis, irMyocarditis and mixed phenotypes

The most frequent presenting symptoms warranting work-up were neurological (myalgia in 4/14 patients and diplopia in 3/14), followed by syncope (2/14), thoracic pain (2/14), fever and fatigue (1/14) and diarrhea (1/14). The 14th patient was initially asymptomatic and was addressed to the hospital after a routine follow-up blood analysis showed elevated CK; he later developed limb-girdle weakness. Median mRs at peak of symptoms was 1.5 (range 1–3).

Clinical phenotypes of limb-girdle skeletal myositis and myocarditis were present, respectively, in 7 (50%) and 10 (71%) patients, either isolated or in combination (mixed phenotype). Seven patients (50%) had ocular involvement (detailed below).

These phenotypes were isolated in most patients (57%) (1 limb-girdle, 4 cardiac, and 3 ocular); the remaining patients had a mixed phenotype (2 limb-girdle + ocular symptoms, 2 limb-girdle + cardiac and 2 with all three phenotypes).

Ocular involvement presented mainly as isolated, non-fluctuating diplopia (6/14), associated in one case to bilateral, asymmetrical non-fluctuating palpebral ptosis. Among the patients with diplopia, three were not diagnosed with ocular myositis during the initial hospitalization: two pointed out the symptom later on to their oncologist, and one was misdiagnosed as 3rd cranial nerve palsy. None of these patients were assessed by a neurologist. Myocarditis diagnosis was considered possible in five patients, probable in one and definite in two. No patient reported symptom fluctuation, muscle fatigability or other clinical hint for myasthenia. Myalgia was a constant symptom in patients with axial or limb weakness. No patient had respiratory symptoms; arterial blood gas analysis was performed and normal in two cases. One patient complained of dysphagia, which required food texture adaptation. Eight patients (57%) developed other autoimmune manifestations, either isolated or combined, including cutaneous irAEs (2/14), dysthyroidism (2/14), colitis (3/14), hepatitis (2/14) and hypophysitis (1/14).

### Biological data

All values peaked during the initial hospitalization. There were elevated levels of CK in 8/15 patients, with wide absolute variations (median 235 units/liter, range 24–7589). All the 12 patients tested for troponin T had abnormally elevated values (median 581 nanograms/liter, range 7–2389). Ultra-specific cardiac troponin-I was tested in nine patients, and was abnormal in seven of them (median 169.9 nanograms/liter, range 28–5896.1). Anti-AChR and anti-MuSK were negative in the only tested patient. Myositis antibody panel was negative in the 4 tested patients as well as 3-hydroxy-3-methylglutaryl-coenzyme A reductase (HMGCR) antibody in patient 4.

### Neurological and neurophysiological assessments

Clinical neurological assessment by the attending physician was performed in all patients, but examination by a neurologist specialist was performed in three patients, and all three were abnormal. Only two patients had a full neurophysiological examination, including nerve conduction studies (motor and sensory conduction in at least two nerves in the upper limbs and two nerves in the lower limbs and 3 Hz repetitive stimulation) which were normal. In both patients, EMG showed abnormal rest potential (fibrillations) and a myogenic pattern upon voluntary activation.

### Imaging and nuclear medicine work-up

**Fig. 1:**
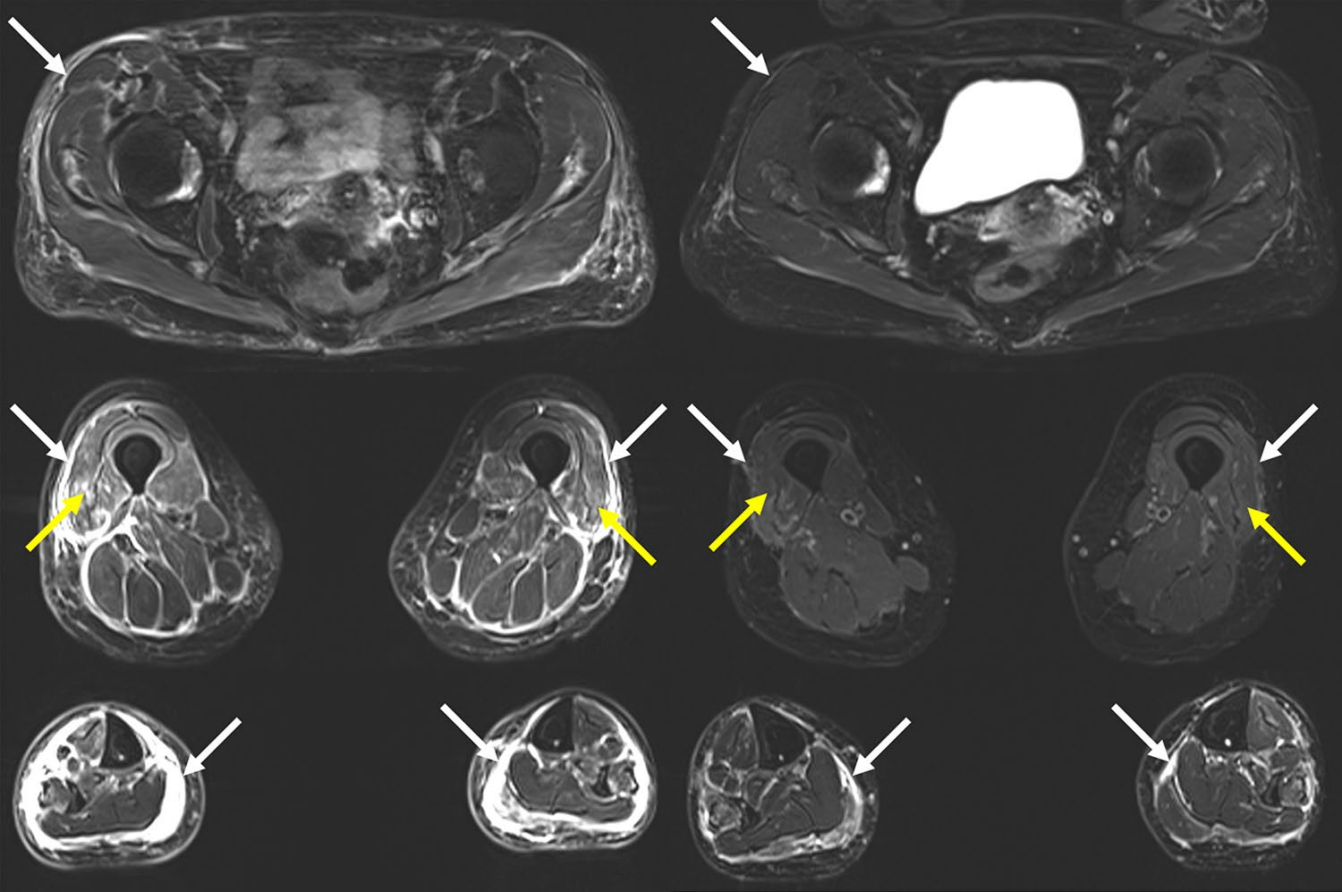
Muscle MRI, patient 2, at time of symptoms (left) and control after 5 months (right). Dixon “water” T2 weighted images, showing symmetrical (with slight right predominance) signs of myositis in vastus lateralis muscles (yellow arrows), and fasciitis (white arrows), predominant in the legs (yellow arrows)

**Fig. 2:**
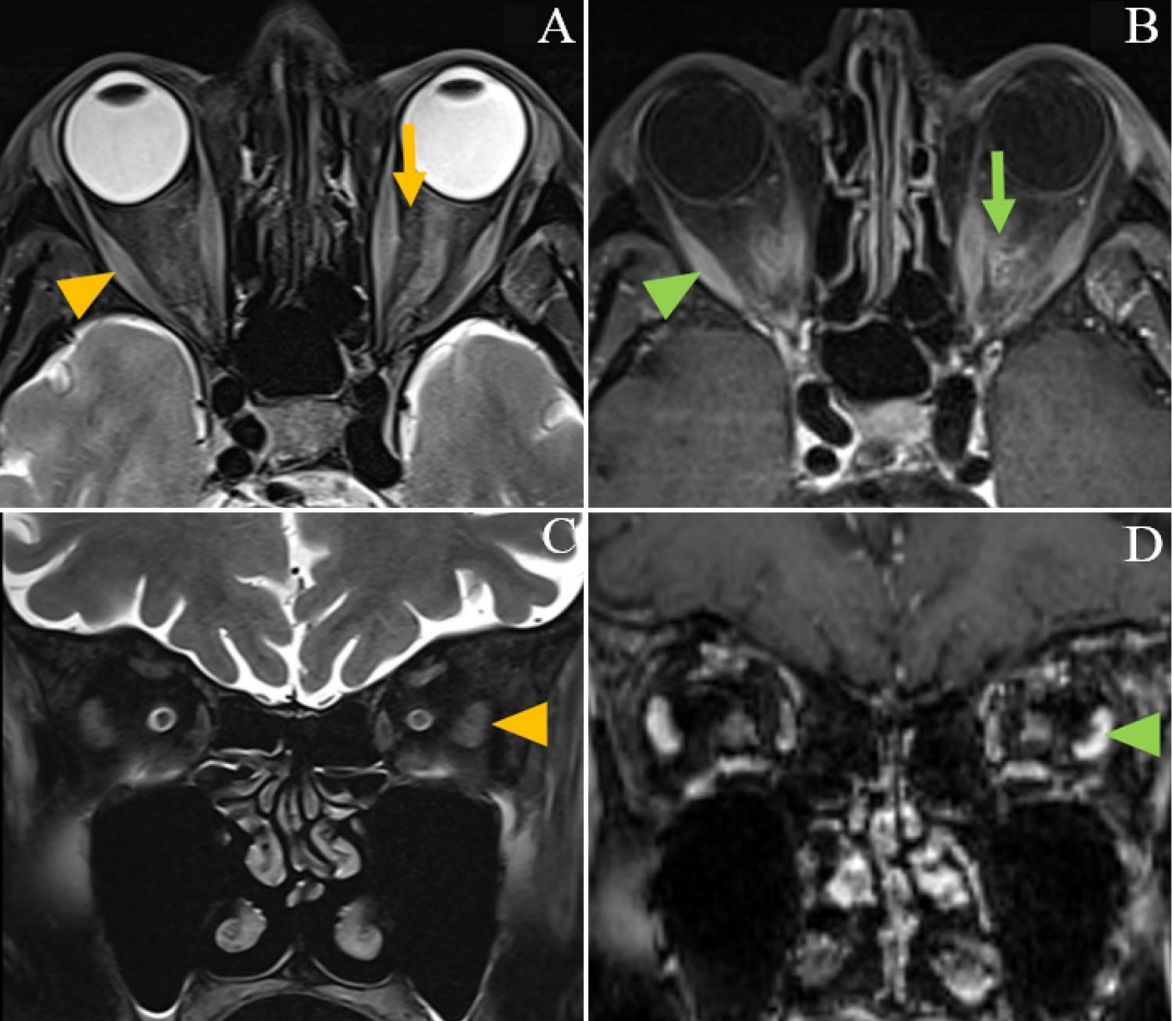
Brain MRI, patients 3 (axial view) and 1 (coronal view). Signs of orbital inflammation. In T2 fat-suppression weighted sequence (**A**, **C**), T2-hypersignal in oculomotor muscle (orange arrowheads) and intraorbital fat (orange arrow). In T1 SPACE 3D fat-saturation weighted sequence (**B**, **D**), presence of muscle (green arrowhead) and intraorbital fat (green arrow) enhancement after gadolinium injection

**Fig. 3:**
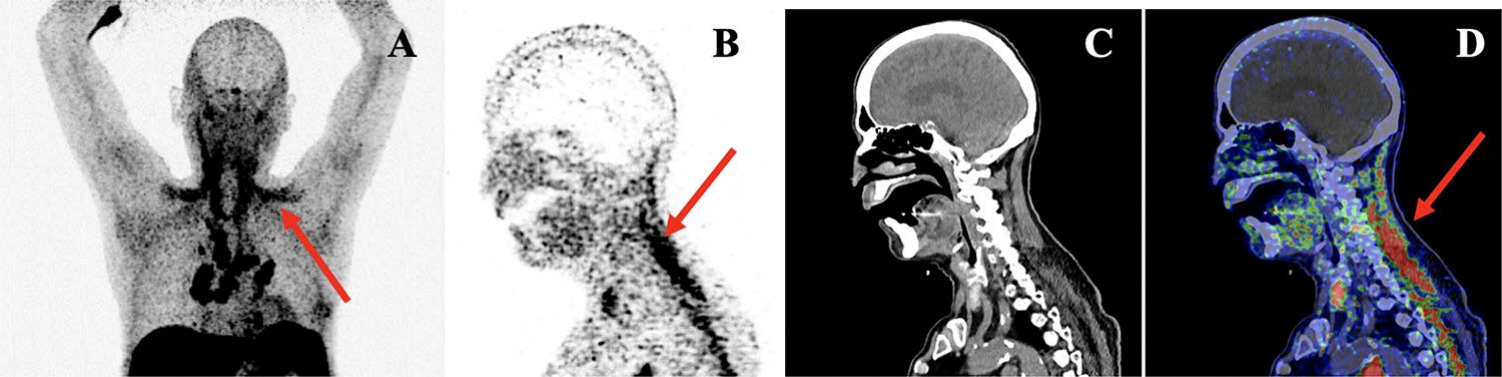
Signs of skeletal myositis on [^68^Ga]-DOTATOC PET/CT scan. Pathological uptake of the trapezius muscle (red arrows) (SUV scale 0–2); **A** Maximum intensity projection imaging. **B** Sagittal PET image, **C** Sagittal CT image and **D** Sagittal fused PET/CT image

**Fig. 4:**
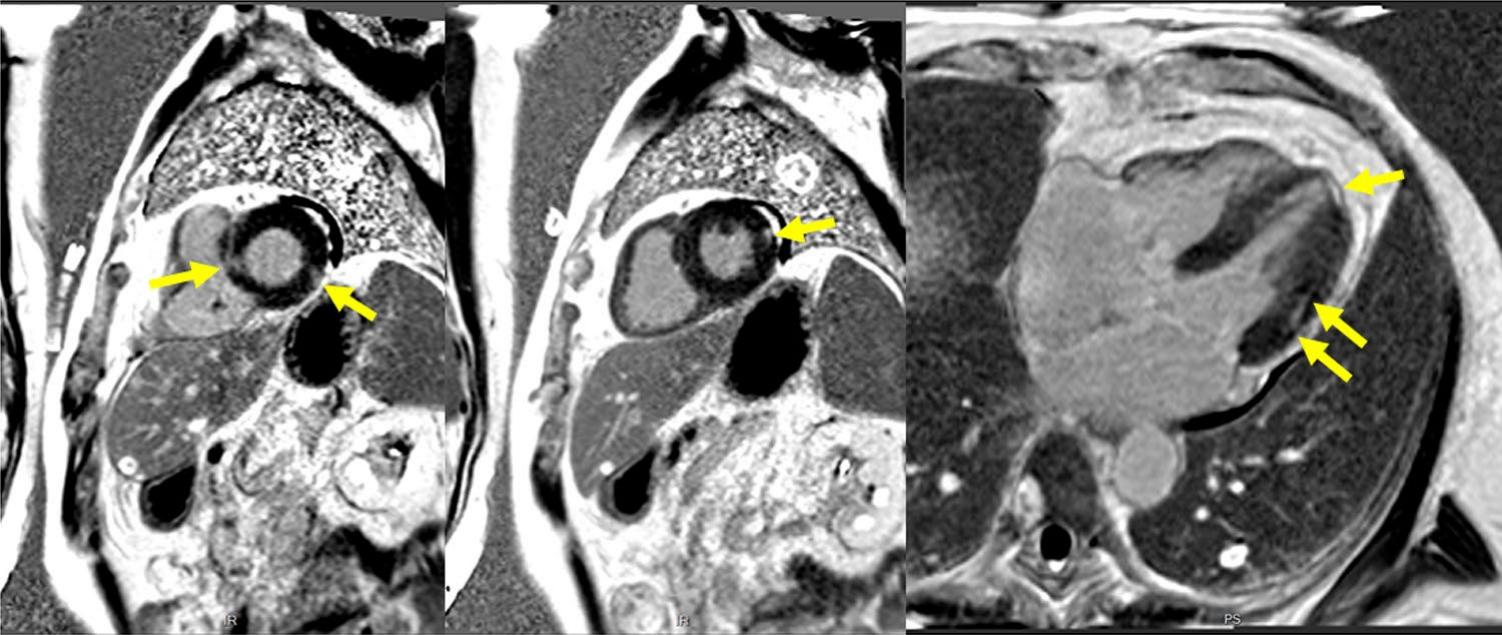
Cardiac MRI, patient 4. Late gadolinium enhancement images obtained with cardiac magnetic resonance in basal and mid-ventricular short axis orientation, and long-axis four-chamber view in a patient with ICI-induced myocarditis. Myocardial enhancement is detected in basal septal, basal inferolateral, mid anterolateral and apical segments with a sub-epicardial distribution (arrows)

There were eight cranial (brain and orbits) MRIs, seven cardiac MRIs and one whole-body muscle MRI. The latter showed signs of myositis and fasciitis in both vastus lateralis muscles, with improvement at 5-month control (Fig. 1). Cranial MRI showed evidence of intra-orbital fat inflammation with gadolinium enhancement in one patient (Fig. 2). After reviewing by a senior neuroradiologist, we identified “missed” signs of myositis (T2 hypersignal) involving upper axial and trapezius muscles in one brain MRI that included the cervical region, with corresponding uptake on PET/CT scan (Fig. 3), and 3 “missed” orbital inflammation signs in other brain MRIs (Fig. 2). In one case, brain MRI showed hypophyseal inflammation, without meningeal involvement. There was evidence of myocarditis with gadolinium enhancement in 6/8 cardiac MRI (Fig. 4). PET-scan showed 10/12 cardiac and 2/12 skeletal muscle uptake of [^68^Ga] Ga-DOTA-TOC (Fig. 3).

### Pathology

**Fig. 5:**
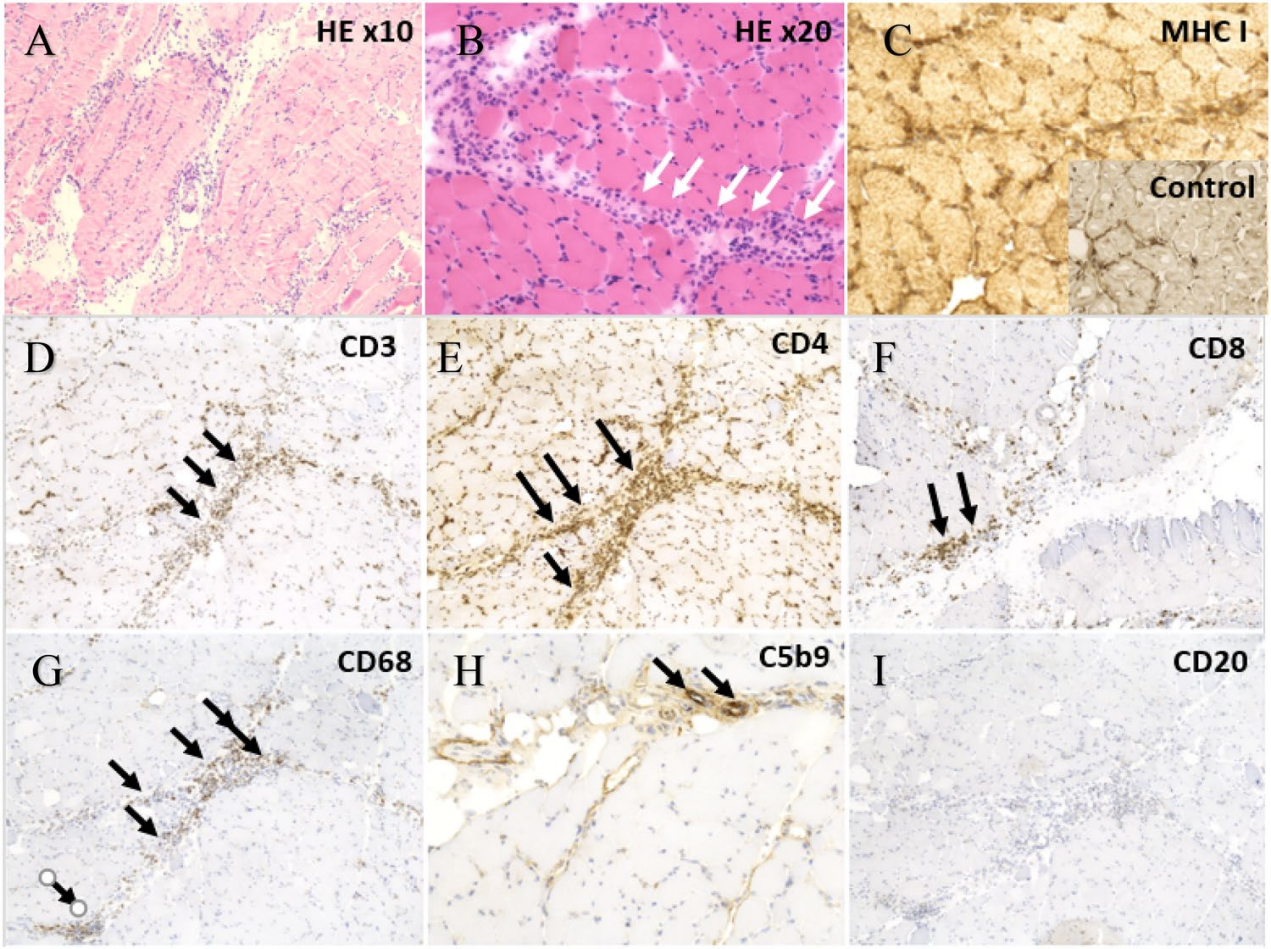
Muscle biopsy, vastus lateralis muscle, patient 2. **A**, **B** hematoxylin and eosin (HE) stain of vastus lateralis muscle, showing abundant, diffuse inflammatory infiltrate predominant in the perimysial area (white arrows). **C** Overexpression of MHC1 in comparison to healthy control (box). Immunohistochemistry showing (**D**) T lymphocytic infiltrate (CD3) of both CD4 and CD8 subtypes (**E**, **F**), macrophage infiltrate (**G**) (CD68) (black arrows). **H** Presence of complement deposition (C5b9, black arrows) in perimysial capillaries. **I** Absence of B lymphocytes (CD20)

Vastus lateralis muscle biopsy (Fig. 5) was performed in patient 2 and showed important endomysial and (predominant) perymysial inflammation, without evidence of necrosis and/or regeneration. The inflammatory infiltrate was composed of CD4 + and CD8 + T lymphocytes, macrophages (CD68 +), without B cells. Traces of complement deposition (C5b-9) were found in the terminal portion of isolated perimysial small vessels. HLA1 (MHC I) was overexpressed in most cells, occasionally with patchy sarcolemmal distribution. There was no evidence of other co-existing underlying muscle pathology. Patient 6 had a myocardial biopsy, which could not demonstrate any inflammatory infiltrate.

### Treatment and outcome

Immunotherapy was discontinued in all patients at diagnosis of irAE. Nine patients received a specific treatment for the irAE, starting with a first-line corticosteroid therapy, intravenous in six patients (methylprednisolone 1 g daily for 3–5 days) or oral in three (prednisone 1 mg/kg daily). Five of the six patients treated with intravenous methylprednisolone secondarily received oral prednisone with gradual dose tapering; after corticotherapy, two patients received a corticoid-sparing immunosuppressant (mycophenolate mofetil). Patient 1, whose initial diagnostic suspicion was myasthenia, received intravenous immunoglobulins (2 g/kg over 5 days) before methylprednisolone. A second-line therapy (one infusion of infliximab 5 mg/kg or tocilizumab 8 mg/kg) was added in patients with myocarditis who had persistent elevation of cardiac biomarkers (troponin T and, when available, I) or a biological relapse despite steroid treatment. Five patients did not receive immunosuppressive treatment. Reasons for therapeutic abstention were co-occurrence of a urinary tract infection and digestive irAE (inflammatory colitis with digestive hemorrhage) in patient 3, and minor symptoms in the others (patients 7, 12, 13, 14), without hemodynamic instability or respiratory failure. These patients were strictly monitored, and ocular and cardiac symptoms improved when immunotherapy was discontinued. No patient needed respiratory support.

Functional (short-term) follow-up median time was 36 days (range 10–60). Outcome was overall favorable: seven patients (50%) had a complete remission of skeletal limb-girdle, ocular and cardiac symptoms and 6 (42%) still had persistent minor symptoms. Biological parameters also globally improved, as showed in Table 2. Median CK decreased from 235 (range 24–7589) to 51.5 U/l (range 18–383), median troponin T value decreased from 581(range 7–2389) to 147 ng/l (range 16–638) and troponin I median value decreased from 169.9 (range 28–5896.1) to 13 (range 3–73.5). Detailed values are reported in supplementary Table 1.

**Table 2:**
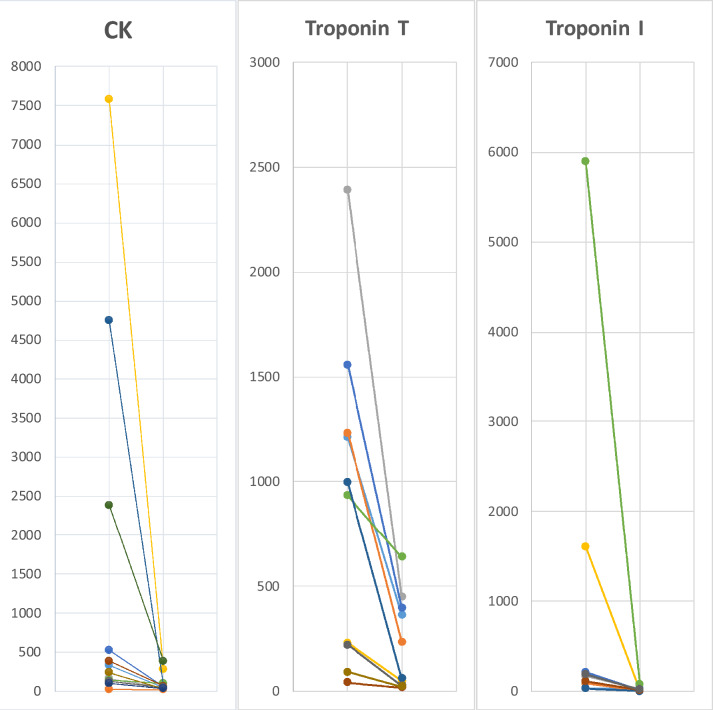
Progression of biological values at T0 and at the end of the follow-up (T1)

CK values are expressed in unit/liters (N: 25–190 U/l) and troponins values in nanograms/liter (troponin T, N < 140 ng/l; troponin I, N < 34.2 ng/l)

Considering long-term outcome, only one patient (n°1) presented with a relapse of myositis-related dysphagia 286 days after the first symptoms, which led to malnutrition and eventually death 354 days after irAE onset. Another (n°6) died 106 days after irAE onset from cancer-related causes, without signs of irAE relapse. Among surviving patients, 4 (28%) were lost to follow-up after a median time of 165 days (range 10–220) without notion of irAE relapse. At our last medical record check (as of August 2023), the remaining followed 8 patients (57%) were relapse-free, after a median follow-up of 3.7 years (range 1.7–5.2).

## Discussion

In this retrospective cohort, we describe 14 consecutive patients who developed irMyositis and /or irMyocarditis. Clinical symptoms occurred within a median time of 2 month after immunotherapy initiation, which is consistent with previous descriptions [15].

Skeletal muscle weakness distribution and clinical presentation were coherent with literature data [10, 15, 16], except for the lower rate of overlap cases, although this result should be considered carefully since most patients did not undergo a complete neurological exam by a specialist, thus leading to a possible underestimation of the prevalence of more subtle neurological symptoms and signs.

Our results show that myalgia is a frequent presenting symptom. It is present in most cases with skeletal and ocular muscle involvement. Therefore, this association might represent a useful hint towards the diagnosis of myositis, especially when considering the differential diagnosis of myasthenia.

Surprisingly, a majority of patients never underwent any neurological examination. The non-homogeneous management probably finds its roots in the lack of knowledge of the characteristic features of this particular irAEs and the absence of diagnostic criteria and guidelines, especially for the first cases in 2018–2019.

Only three patients underwent a full neuromuscular assessment, and neurophysiological assessment was performed in only two. As a possible consequence, three co-existing ocular myositis (which are pathognomonic of irAE myositis) were undiagnosed (2/3) or misdiagnosed (1/3, as 3rd cranial nerve palsy). Interestingly, after brain MRI review, we also identified three previously undiagnosed cases of orbital inflammation. Our results confirm the high prevalence of ocular muscle involvement, which is typical of this kind of myositis and thus an important clue to diagnosis, but which can however easily be missed.

Evidence shows that muscle irAE complications might encompass a spectrum including skeletal myositis, myocarditis and to some extent, myasthenia [10]. Up to a quarter of all subjects with myocarditis also presents with skeletal myositis [17] and myasthenia [18], although in the less recent literature, some ocular or pharyngeal myositis might have possibly been misdiagnosed as myasthenia. While myositis is induced by ICI, some evidence points out that myasthenic syndrome can be subclinically pre-existing and unveiled by the autoimmune activation [19–21]. Since steroid treatment and especially oral prednisone [22] can lead to paradoxical exacerbation of muscle and respiratory failure in myasthenia, this diagnosis has direct implications for monitoring (including respiratory assessment of forced vital capacity). Conversely, the exclusion of myasthenia in a patient with limited ocular myositis might influence the discussion about ICI reintroduction, especially for patients with few or no other cancer therapeutic option. Moreover, other neurological irAEs are described, affecting both the peripheral and central nervous system [10]. Some complications, in particular acute inflammatory neuropathy, are susceptible of potentially lethal complication as respiratory failure and dysautonomia [23]. Therefore, we would highly recommend an assessment by a neuromuscular specialist with electrodiagnostic studies, even in patients with apparently isolated cardiomyopathy.

Out results confirm the importance of biological assessment, with almost-invariably elevated CK levels. We stretch the importance of dosing cardiac specific troponins I to increase specificity and help the assessment of response to treatment in case of myocarditis [24].

Given the high frequency of skeletal and myocardial muscle co-involvement, we recommend to perform electrocardiogram and troponin T in all patients with skeletal or ocular irMyositis even in the absence of cardiac symptoms, to be completed if needed with advanced tests [25]. Conversely, as previously stated, all patients with isolated irMyocarditis should undergo a neuromuscular clinic assessment and neurophysiological studies, since skeletal muscle co-involvement can worsen the prognosis by increasing the risk of respiratory complications [10].

Muscle biopsy was only performed in the first diagnosed case and deemed unnecessary in other patients with a typical clinical and biological presentation after immunotherapy introduction. As previously described [15], we did not find any CD20 cellular infiltrate but a rich macrophage (CD68) and T lymphocytic (CD4 + and CD8 +) infiltrate, as well as a diffuse overexpression of MHC I. Inflammatory changes were predominantly perimysial, in line with more recent descriptions [26].

Imaging techniques play a major role in the work-up of irMyositis and irMyocarditis patients.

The role of MRI is well known in irMyocarditis diagnosis and it was abnormal in about a half of irMyocarditis in our cohort, a slightly lower rate than described in international recommendations. As previously mentioned, brain and muscle MRI helped unveiling ocular and skeletal myositis signs, in some cases previously undiagnosed, suggesting a role in detecting subclinical involvement.

Although not included in irMyocarditis diagnostic criteria at the time of the study, [^68^Ga]-DOTATOC PET/CT is potentially interesting for the diagnosis of irAE cardiac complications [27]. Its use is now recommended in recent ESMO guidelines [6]. Some evidence pointed out the interest of this marker (selective to type 2 and type 5 somatostatin receptors) in cardiac inflammation, especially on activated T cells [28]. Radiological work-up showed some discordance between MRI and PET/CT scan findings in our study; this might suggest a higher sensitivity of the latter, especially in the first stages of inflammation, when cardiac edema and inflammatory changes might not yet be visible on the MRI, but it could also suggest a lower specificity. Our findings also suggest a potential interest of the technique for the detection of skeletal myositis, which has not been previously reported in the literature.

Finally, in our cohort, functional outcome was generally good following appropriate treatment, including immunotherapy suspension, corticosteroid treatment and, when indicated, immunosuppressant as suggested for other complications [29].

Our data also show a globally positive long-term outcome in terms of relapse after immunosuppressant tapering and overall survival.

We acknowledge the obvious limitations related to the retrospective and real-world nature of data; diagnostic work-up were tailored on patients’ clinical presentations, influencing data availability for each patient.

## Conclusion

irMyositis is a potentially serious immune-related adverse event, recognizable by specific clinical and biological features. Despite being known for more than 4 years, the potentially mischievous presentation can expose the patients to misdiagnosis. In addition to confirming previously described clinical features of irMyositis, our research adds interesting results about clinical presentation, diagnostic pitfalls, imaging contribution and long-term outcome.

Recognition of wider muscle involvement (including myalgia and ocular signs) and avoidance of misdiagnosis (e.g. of myasthenia), can have direct implication on management, monitoring during immunosuppressive treatment introduction, and outcome.

We recommend that a neuromuscular assessment is systematically performed, even when myocarditis is the only apparent presentation, and that reference centers train a multi-disciplinary task force in order to quickly detect irAEs, covering all the facets of these complex syndromes and harmonize investigations and treatment.

### Supplementary Information

Below is the link to the electronic supplementary material.

Supplementary file1 (DOCX 68 KB)
