# Supplementary material for: Immune checkpoint inhibitor-related myositis and myocarditis: diagnostic pitfalls and imaging contribution in a real-world, institutional case series
Source: J Neurol. 2023 Dec 23;271(4):1947–58. doi: 10.1007/s00415-023-12134-x (PMC10973051; doi:10.1007/s00415-023-12134-x)
Supplement: Supplementary file 1 — Supplementary file1 (DOCX 68 KB) [file 415_2023_12134_MOESM1_ESM.docx]

# Supplementary material for

“Vicino et al - Immune checkpoint inhibitor-related myositis and myocarditis: diagnostic pitfalls and imaging contribution in a real-world, institutional case series”

| **N°** | **CK** | | **Troponin T** | | **Troponin I** | |
| --- | --- | --- | --- | --- | --- | --- |
|  | **T0** | **T1** | **T0** | **T1** | **T0** | **T1** |
| 1 | **331** | 46 | **1210** | **363** | 30.7 | 27.5 |
| 2 | 24 | 18 | **18** | n/a | n/a | n/a |
| 3 | 26 | n/a | n/a | n/a | n/a | n/a |
| 4 | 158 | 46 | 7 | n/a | n/a | n/a |
| 5 | **7589** | **275** | **1231** | **235** | **88.7** | 4.9 |
| 6 | **524** | 56 | **2389** | **448** | **169.9** | 23.9 |
| 7 | 142 | 99 | **229** | **49** | n/a | n/a |
| 8 | **4752** | 65 | **1556** | **397** | **1604.7** | 6 |
| 9 | **386** | 68 | **933** | **638** | **212** | 10 |
| 10 | 127 | 47 | **996** | **59** | **5896.1** | **73.5** |
| 11 | **235** | 31 | **40** | **16** | 28 | 3 |
| 12 | 101 | 33 | **219** | **26** | **110** | 13 |
| 13 | **2385** | **383** | **91** | **22** | **187** | 15 |
| 14 | n/a | n/a | n/a | n/a | n/a | n/a |

*Supplementary table 1. Biological parameters progression over the observation period.*

Abnormal biological values are highlighted in bold.

CK: creatinine kinase (N: 25-190 Units/liter). Troponin T (N<14 nanograms/liter). Troponin-I (N<34.2 nanograms/liter).
